# Union Medical Association

**Published:** 1856-09

**Authors:** G. W. Hotchkiss, W. Duff Green

**Affiliations:** Pres.; Sec’y.


*Union Medical Association.*

Pursuant to a call through the public press, the physicians of Southern Illinois met at Centralia, on the 1st day of April, for the purpose of organizing a Medical Society auxiliary to the State Medical Society of Illinois.

F. B. Haller, M.D., of Vandalia, was chosen Chairman of the meeting, and W. D. Green, M.D., of Mt. Vernon, Secretary.

The Society to be known as the UNION MEDICAL ASSOCIATION, of Southern Illinois.

The Association adopted a Constitution and a Code of By-Laws, also, a Tariff of Charges for the government of its members.

The following gentlemen were elected officers for the ensuing year:—

George W. Hotchkiss, M.D., *President.*

F. B. Haller, M.D., *Vice-President.*
W. D. Green, M.D., *Secretary.*
Wm. White, M.D., *Treasurer.*
A. D. Stearns, M.D., }
H. C. Winans, M.D., } *Censors.*
T. Wilkins, M.D., }

The meetings of the Association to be semi-annual, on the third Tuesday of May and the second Tuesday of November.

The next meeting to be at Salem, Ill., Tuesday, November 11th, 1856.

G. W. HOTCHKISS, M.D., *Pres.*
W. DUFF GREEN, M.D., *Sec'y.*

